# Exosomal miR-224-3p promotes lymphangiogenesis and lymph node metastasis by targeting GSK3B in gastric cancer

**DOI:** 10.32604/or.2024.050431

**Published:** 2025-01-16

**Authors:** ZHENGYANG ZHOU, LEI QIAO, TONGTONG WANG, WEN PAN, JINGJING DUAN, HAIYANG ZHANG, TING DENG, YI BA, YI HE

**Affiliations:** 1 Tianjin’s Clinical Research Center for Cancer, Tianjin Medical University Cancer Institute and Hospital, Tianjin Medical University, Tianjin, 300060, China; 2 Tianjin Union Medical Center of Nankai University, Nankai University, Tianjin, 300060, China

**Keywords:** Gastric cancer (GC), Extracellular vesicles (EVs), miR-224-3p, Lymph node metastasis (LNM), GSK3B

## Abstract

**Background:**

Patients with gastric cancer (GC) are prone to lymph node metastasis (LNM), which is an important factor for recurrence and poor prognosis of GC. Nowadays, more and more studies have confirmed that exosomes can participate in tumor lymphangiogenesis. An in-depth exploration of the pathological mechanism in the process of LNM in GC may provide effective targets and improve the diagnosis and treatment effect.

**Materials and Methods:**

We used sequencing analysis of collected serum to screen out exo-miRNA related to LNM in GC. ELISA, qRT-PCR, Western Blot, RNA pull-down assay, Transwell assay, animal experiments, and other experiments were used to verify the results.

**Results:**

In this study, we screened out miR-224-3p related to GC progression and LNM in a vascular endothelial growth Factor C (VEGFC)-independent manner. We found that exo-miR-224-3p derived from GC cells could enter human lymphatic endothelial cells (HLECs) and promote the tube formation and migration of HLECs. In addition, it was revealed that miR-224-3p could bind to the 3′UTR region of GSK3B mRNA. Then, we proved that inhibiting the expression of GSK3B could suppress the phosphorylation of β-catenin and promote the transcription of PROX1, thus leading to tumor lymphangiogenesis. Furthermore, it was also found that hnRNPA1 mediated the sorting of miR-224-3p into exosomes, and the high expression of PKM2 promoted the secretion of exo-miR-224-3p.

**Conclusions:**

Our discovery of the exo-miR-224-3p/GSK3B/β-catenin/PROX1 axis may provide a new direction for the clinical treatment of GC.

## Introduction

Gastric cancer (GC) is a malignant tumor with a high incidence worldwide that seriously affects human health [1,2]. Lymph node metastasis (LNM) in patients with GC usually indicates a poor prognosis [3,4]. How to effectively intervene in LNM in GC may improve the treatment effect for patients. LNM in GC is a complex and orderly process involving the acquisition of an invasive phenotype at the primary tumor site, preparation of metastasis channels, regulation of the immune microenvironment, preparation of the microenvironment at the metastasis site, and other pathological processes [5–8]. In-depth exploration and study of the pathological mechanism in the process of LNM would allow for a better understanding of the biological behavior of GC and provide more targets for diagnosis and treatment [9,10].

Extracellular vesicles (EVs) are a class of microvesicles ranging in size from 30 to 150 nm that carry a large amount of parental cell information and contain various small molecular substances, such as proteins, lipids, and miRNAs [11]. It has been reported that exo-miR-548k could promote vascular endothelial growth Factor C (VEGFC) secretion and then increase the formation of lymph nodes by regulating the ADAMTS1/VEGFC/VEGFR-3 pathway [12]. This process is achieved between cells through the delivery of EVs. EVs secreted by tumor cells, as crucial messengers of intercellular communication, are involved in immune microenvironment suppression, tumor-associated angiogenesis, lymphangiogenesis, and other pathological processes [13–17]. There was research suggesting that sumoylation in hepatocellular carcinoma cells promotes PKM2 to target the plasma membrane in the form of microvesicles and then promotes tumor progression by inducing monocytes to differentiate into M2-like macrophages [18]. However, the mechanism by which EVs derived from tumor cells regulate lymphangiogenesis and LNM still needs to be further explored.

RNA-binding proteins (RBPs) are key regulators in posttranscriptional processing and RNA molecular regulation. The interaction between RBPs and multivesicular body (MVB) components makes it possible for RBPs to participate in the sorting of exosomal miRNAs [19]. In the cell, miRNA can be specifically sorted into EVs via RBPs and enter the circulation system to achieve communication between cells and distant cells or tissues [19,20]. It has been confirmed that miR-196a and miR-522 can be loaded into fibroblast EVs by hnRNPA1. The former leads to cisplatin chemotherapy resistance in head and neck tumors, while the latter is secreted by tumor-associated fibroblasts and taken up by GC cells to inhibit ferroptosis, causing drug resistance [21,22]. Therefore, the study of miRNA sorting into EVs may help us better understand the communication between tumor cells and other cells and contribute to developing therapeutic targets against secreted exo-miRNAs.

Glycogen synthase kinase-3 (GSK-3), an evolutionarily conserved serine/threonine kinase in mammals, was first reported in 1980 [23]. Mammals express two GSK-3 isoenzymes, GSK3B (47 kDa) and GSK3 (51 kDa), which are encoded by different genes and contain 97% sequence similarity in the kinase domain. However, these isoenzymes are completely different outside the kinase domain [24]. Inhibition of GSK-3B could reduce the phosphorylation of β-catenin, thereby preventing the degradation of β-catenin. Stable β-catenin accumulates in the cytoplasm and is transported to the nucleus via Rac1 and other factors [25]. The β-catenin signaling pathway is activated to promote the transcription of downstream target genes. These studies indicate that GSK3B plays an important role in the process of tumor lymphangiogenesis and LNM, while the specific regulatory mechanism remains to be further explored.

In this study, we used serum miRNA sequencing data from GC patients and found that miR-224-3p was upregulated in the serum of patients with LNM. The role of exo-miR-224-3p in promoting lymphangiogenesis and metastasis was revealed *in vitro* and *in vivo*. In addition, GSK3B was confirmed to be a direct target of miR-224-3p. Our results demonstrate, for the first time, that miR-224-3p is loaded into EVs mediated by hnRNPA1 and that exo-miR-224-3p promotes tumor lymphangiogenesis and LNM through the GSK3B/β-catenin/PROX1 pathway. These findings may provide potential biomarkers and targets for the diagnosis and treatment of LNM in GC.

## Materials and Methods

### Serum and tissue samples from clinical GC patients

A total of 65 serum samples were collected from GC patients at Tianjin Medical University Cancer Institute Hospital from June 2020 to June 2021, including 38 patients with LNM and 27 patients without LNM. All patients had a pathological diagnosis of GC and had not received any tumor-related treatment before sampling. All peripheral blood samples were drawn in the morning under fasting conditions, and venous blood was collected in anticoagulant tubes and centrifuged at 800× *g* for 15 min. After centrifugation, the supernatant was placed in an enzyme-free EP tube and stored at −80°C or in liquid nitrogen. A total of 32 pairs of tissue specimens and paracancerous tissues (more than 5 cm from the edge of the tumor were collected, including 14 pairs of samples without LNM and 18 pairs of samples with LNM. All of the above patients were informed of the contents of this study and consented to the experiment. In addition, experimental ethics were approved by the Ethics Committee of Tianjin Medical University Cancer Institute and Hospital (No. Ek2021002).

### GEO data download and analysis

In the GEO database (https://www.ncbi.nlm.nih.gov/geo/, accessed on 01/05/2024), miRNAs in gastric cancer of miRNAs were used as the keyword search for serum miRNA GC datasets, and all miRNA expression data and clinical information were downloaded. The miRNA expression data and clinical information of GC-related serum and healthy control (HC) serum were screened for subsequent GEO2R analysis. The GC and HC serum extracted from GSE112264 (GC *vs*. HC) and GSE85589 (GC *vs*. HC) was analyzed by GEO2R, and the *p*-value < 0.05, |log_2_ FC| > 1.

### Serum miRNA sequencing analysis

We collected serum samples from 24 newly diagnosed GC patients (without any treatment) at Tianjin Medical University Cancer Institute Hospital, including 12 patients with positive LNM and 12 patients with negative LNM (nonLNM). Serum miRNA sequencing was performed by Lianchuan Biotechnology Co., Ltd. (Hangzhou, China). We used the R package DESeq2 to perform differential analysis of serum data (LNM *vs*. nonLNM), and *p*-value < 0.05, |log_2_ FC| > 1.

### Enzyme-linked immunosorbent assay (ELISA)

The collected serum was removed from the −80°C refrigerator and thawed on ice. The samples were centrifuged at 800× *g* for 15 min at 4°C. The remaining steps of serum vascular endothelial growth Factor C (VEGFC) detection were performed according to the ELISA kit protocol (Elabscience, Wuhan, China).

### Cell culture

Six cell lines were used in this study, including the human gastric cancer cell lines HGC27, MKN45, and AGS, the human primary lymphatic endothelial cell line HLECs, the normal gastric epithelial cell line GES-1, and the human embryonic kidney cell line HEK-293FT. All the above cells were purchased from the Shanghai Cell Bank, Chinese Academy of Sciences (Shanghai, China). Five percent FBS-ECM medium was used for HLECs, 20% FBS-RPMI-1640 medium was used for HGC27 cells, and 10% FBS-RPMI-1640 medium was used for MKN45 cells. Ten percent FBS-DMEM was used for GES-1 and HEK293-FT cells, and 10% FBS-F12 medium was used for AGS cells. All cells were cultured in a 5% CO_2_, 37°C constant temperature incubator and moisturized with water.

### Protein extraction and western blotting

Protein was isolated from cultured cells and tissues using sodium dodecyl sulfate (SDS) lysis buffer (Solarbio, Beijing, China) with a protease inhibitor freshly added. After that, the lysates were separated via SDS‒PAGE gels and transferred onto polyvinylidene fluoride (PVDF) membranes. The immunoblots were blocked with 5% skim milk (TBST prepared) at room temperature for 1 h and incubated at 4°C overnight with anti-hnRNPA1 (Abcam, Cambridge, UK), anti-PKM2 (Abcam, UK), anti-Ki67 (Santa Cruz Biotechnology, Dallas, TX, USA), anti-CD9 (Abcam, UK), anti-TSG101 (Santa Cruz Biotechnology, USA), and anti-CD63 (CST, Danvers, MA, USA) antibodies. Following incubation with the proper secondary antibodies (Santa Cruz Biotechnology, USA), the membranes were visualized with an enhanced chemiluminescence kit according to the manufacturer’s protocol.

### Extraction of serum RNA

The collected serum was taken from a −80°C refrigerator and thawed on ice. The samples were centrifuged at 800× *g* for 15 min at 4°C. Then, 300 μL of supernatant was placed in a sterile, enzyme-free 1.5 mLEP tube, and 200 μL of DNase/RNase-Free Water (DDW) was added. Then, 250 μL of acid phenol was added to the EP tube, shaken vigorously, left at room temperature for 2 min, and centrifuged at 16000× *g* for 15 min at 4°C. Approximately 400 μL of supernatant was added to a new 1.5 mL sterile EP tube, 1/10 volume (approximately 40 μL) of sodium acetate (3 M, pH = 5.2) was added, and the mixture was mixed thoroughly. Then, twice the volume of supernatant (approximately 800 μL) of isopropanol was added and left at −20°C for 60 min. Then, it was centrifuged at 16000× *g* for 20 min at 4°C. The supernatant was discarded, and small white spots were observed. Next, 75% ethanol precooled at −20°C was added to the white spot, which was then centrifuged at 16000× *g* for 20 min at 4°C. The supernatant was discarded and allowed to dry for 10–20 min until the small white spots became transparent. An appropriate amount of DDW was added according to the size of the small white dots. RNA concentration was measured by Nanodrop 2000, which could be stored at −80°C for later use.

### Extraction of serum EVs (Ultracentrifugation)

The collected serum was taken from a −80°C refrigerator and thawed on ice. Serum was added to a 15 mL centrifuge tube, balanced with an equal volume of phosphate-buffered saline (PBS), and centrifuged at 2000× *g* for 30 min at 4°C. The supernatant was transferred to a new 15 mL centrifuge tube, balanced, and centrifuged at 10000× *g* for 45 min at 4°C. The supernatant was filtered through a 0.22 μM filter and then transferred into the supernatant tube, balanced, and centrifuged at 100000× *g* for 4 h at 4°C. The supernatant was taken, resuspended in 1 mL of PBS, added to 12 mL of PBS (according to the specific volume of the ultracentrifugation tube), balanced, and centrifuged at 100000× *g* for 2 h at 4°C. The supernatant was discarded, and the cells were resuspended in 1 mL of PBS. Then, 12 mL of PBS was added, balanced, and centrifuged at 100000× *g* for 2 h at 4°C. The supernatant was discarded, emptied as much as possible, and then resuspended by adding approximately 50 μL of PBS.

### Size distribution and particle concentration measurement of sEVs

The size distribution and particle concentration of sEVs were measured by using a nano flow cytometer (N30E Nanoflow Analyser, NanoFCM Inc., Xiamen, China) at EchoBiotech Co., Ltd., Beijing, China. Briefly, the side scatter intensity (SSI) was measured by loading standard polystyrene nanoparticles (250 nm) into the nanoflow cytometer. Next, isolated sEV samples diluted with PBS (according to the BCA protein assay results, the EVs were diluted to 1–10 ng/μL) were loaded onto the nanoflow to measure the SSI. Finally, the concentration of EVs was calculated according to the ratio of SSI to particle concentration in the standard polystyrene nanoparticles. For size measurement, standard silica nanoparticles with mixed sizes (68, 91, 113, and 155 nm) were loaded onto the nano flow cytometer to generate a standard cure, followed by the loading of sEV samples. The size distribution was calculated according to the standard cure.

### Quantitative detection of miRNAs

The reverse transcription system was prepared according to the table below and operated on ice. The reagent system was as follows: 5 × M-MLV buffer for 4 μL, M-MLV reverse transcriptase for 1 μL, dNTP mixture for 2 μL, reverse transcription primers (miRNA/U6) for 2 μL, RNA for X (2 μg), DDW fill to 20 μL. After the addition of the above components, the samples were mixed and centrifuged at 5000 rpm and 4°C for 2 min. Reverse transcription was performed according to the procedures in the following order: 16°C for 30 min, 42°C for 30 min, 85°C for 5 min, and 4°C for cycles. Then, the quantitative real-time PCR (qRT-PCR) system was prepared according to the table below and operated on ice away from light. The reagent system was as follows: 2 × SYBR GREEN mix for 10 μL, cDNA for 1 μL, forward primer (5 μM) for 0.8 μL, reverse primer (5 μM) for 0.8 μL, and DDW for 7.4 μL. After the addition of the above components, they were mixed and centrifuged at 5000 rpm for 2 min at 4°C. Then, the plates were placed into 96-well plates in turn, sealed, and centrifuged at low speed (800 rpm) and dark room temperature for 3 min. QRT‒PCR was performed according to the following procedure: 95°C for 10 min, 95°C for 2 s, 60°C for 20 s, 70°C for 30 s, 70°C–95°C for 5 s/time, and 4°C for cycles. The Ct value of each sample was obtained after qRT‒PCR. Expression = 2^−Δt^, and Δt = Ct (sample) – Ct (the internal reference). The internal reference used for miRNA detection in this study was U6.

### Prediction of RBPs by the RBPDB database

We used the RBPDB database (Database of RBP usage, http://rbpdb.ccbr.utoronto.ca/, accessed on 01/05/2024) to analyze the specific interaction between the miR-224-3p sequence and RNA binding protein (RBP), with thresholds of 0.5 and score >5.

### RNA pull-down assay

NP40 lysates were mixed with PMSF (1:100) and RNase inhibitor (1:1000) to prepare RNA pull-down cell lysates. One milliliter of the prepared cell lysate was added to a 10 cm petri dish with a cell density of 90% and lysed for 30 min on ice. Cell lysates were collected in enzyme-free 1.5 mL EP tubes and centrifuged at 2000× *g* for 10 min at 4°C. The supernatant was removed and centrifuged at 10000× *g* for 10 min at 4°C. After that, the supernatant was collected and divided into four equal portions for later use. With one copy as input, 500 pmol of biotin-modified miRNA (bio-Wnt), biotin-modified mutated RBP binding region miRNA (bio-Mut) or biotin-modified whole G Negative control sequence (bionegative) was added to the remaining three. The cells were incubated overnight (>12 h) at 4°C on a shaker. Streptavidin magnetic beads (SA beads, 500 μL) were placed on a magnetic rack for 1 min, the storage solution was discarded, and the magnetic rack was washed with 1× PBS twice for 1 min each. The treated SA beads were added to each overnight incubated fraction, mixed, and incubated for 4–6 h at 4°C on a shaker. After incubation, SA beads were washed with 1× PBS 5 times, and a magnetic rack was used for 1 min each time. The PBS was removed, 80–100 μL of SDS-containing lysate was added, and the mixture was boiled for 10 min at 100°C. After cooling, the mixture was placed in a magnetic rack for 3–5 min, and the supernatant protein was absorbed to measure the concentration by the BCA method.

### Coculture of EVs and cells

The cells were spread into 6-well plates one day in advance, and it was expected to reach a density of 65%–75% the next day (6-well plate as an example). The medium was discarded, and after two washes with 1× PBS, 5% fetal bovine serum (FBS) complete medium with serum excluding EVs was added, and 100 μg EVs were added to each well for coculture with cells. Subsequent experiments could be performed after 24–48 h.

### Transwell assay

The treated cells were digested, and the cell concentration was adjusted to 10^6^/mL using the conditioned medium with 5% FBS. Then, 400 μL of medium with 15% FBS was added to the lower chamber, and 200 μL of cell suspension was added to the upper chamber and mixed with light shaking. The culture plate was placed in the cell incubator, and the time was selected according to the cell line (HLEC perforation time was 16–20 h). After the culture was completed, the chamber was removed, and the medium in the upper chamber was discarded. The cells were washed once with 1× PBS and fixed in methanol for 15 min. After that, the cells in the upper chamber were gently wiped off with a cotton swab, and after washing 3 times with 1× PBS, the lower chamber was immersed in 0.1% crystal violet solution and stained for 15 min. The stained chambers were cleaned and dried with tap water, and the membrane at the bottom of the chamber was gently cut with a knife. The cells were spread on clean glass slides with the cell face up, sealed with medium resin, and dried in a ventilation cabinet overnight. The next day, the results were observed under a microscope and photographed.

### In vitro human tube formation assay

Matrigel (Corning, NY, USA) was removed and placed on ice overnight to thaw the sample the day before the experiment. The 24-well plate, autoclaved gun head, and EP tube were precooled at −20°C in advance, and the operation of Matrigel involved in the whole experiment was carried out in an ice box. The melted Matrigel with a high concentration was diluted 1:1 with basal medium. Matrigel (200 μL) was uniformly spread in a precooled 24-well plate. Be careful not to create bubbles. The Matrigel solidified after the 24-well plate was placed in the incubator at 37°C for 30–45 min. After digestion and centrifugation, HLECs were counted. A 5 × 10^5^/200 μL of cell suspension was slowly added to the 24-well plate, and the cells were spread with gentle shaking. The 24-well plates were returned to the incubator for further incubation for 4–8 h. After that, the plates were observed under a microscope and photographed to record the formation of tubes.

### Animal experiments

All BALB/c nude were purchased from GemPharmatech (Jiangsu, China).The ethics of animal experiments were reviewed and approved by the Animal Experiment Management Committee of Tianjin Medical University Cancer Institute and Hospital (Approval number: NSFC-AE-2021002). The constructed MKN45-Luci-OE, MKN45-Luci-SP, and MKN45-Luci-NC cells were implanted into the foot pads of nude mice (n = 5 in each group). The basic status of the nude mice and the growth of tumors on the foot pads were recorded every three days. After 6–8 weeks, luciferase substrate was intraperitoneally injected into nude mice, and *in vivo* imaging was performed to observe tumor metastasis. When the size of tumors on foot pads grew to more than 200 mm^3^, the nude mice were sacrificed. Meanwhile, the *in situ* and popliteal lymph nodes of nude mice were removed (Exo-miR-224-3p derived from GC cells could promote LNM *in vivo*). The constructed MKN45-Luci cells were implanted subcutaneously in nude mice. One week later, the basic condition and tumor growth of the nude mice were observed. When the volume of subcutaneous tumors reached approximately 20 mm^3^, nude mice were randomly divided into two groups (n = 6 in each group). In the first group, EVs with high expression of miR-224-3p (MKN45/miR-224-3p-exo) were injected around the transplanted tumors, and in the second group, normal control EVs were injected around the transplanted tumors (MKN45/NC-exo). Tumor growth was measured and recorded every three days. When the tumor grew to more than 250 mm^3^ *in situ*, *in vivo* imaging of small animals was performed to detect these tumors (Exo-miR-224-3p derived from GC cells could promote lymphangiogenesis *in vivo*).

### Statistical analyses

All of the data are representative of at least three independent experiments and are expressed as the mean ± SE. A *p*-value < 0.05 was considered to be statistically significant using Student’s *t*-tests: *p-value < 0.05; **p-value < 0.01; and ***p-value < 0.001; ****p-value < 0.0001.

## Results

### Exosomal miR-224 was screened to be associated with LNM of GC

**Figure 1:**
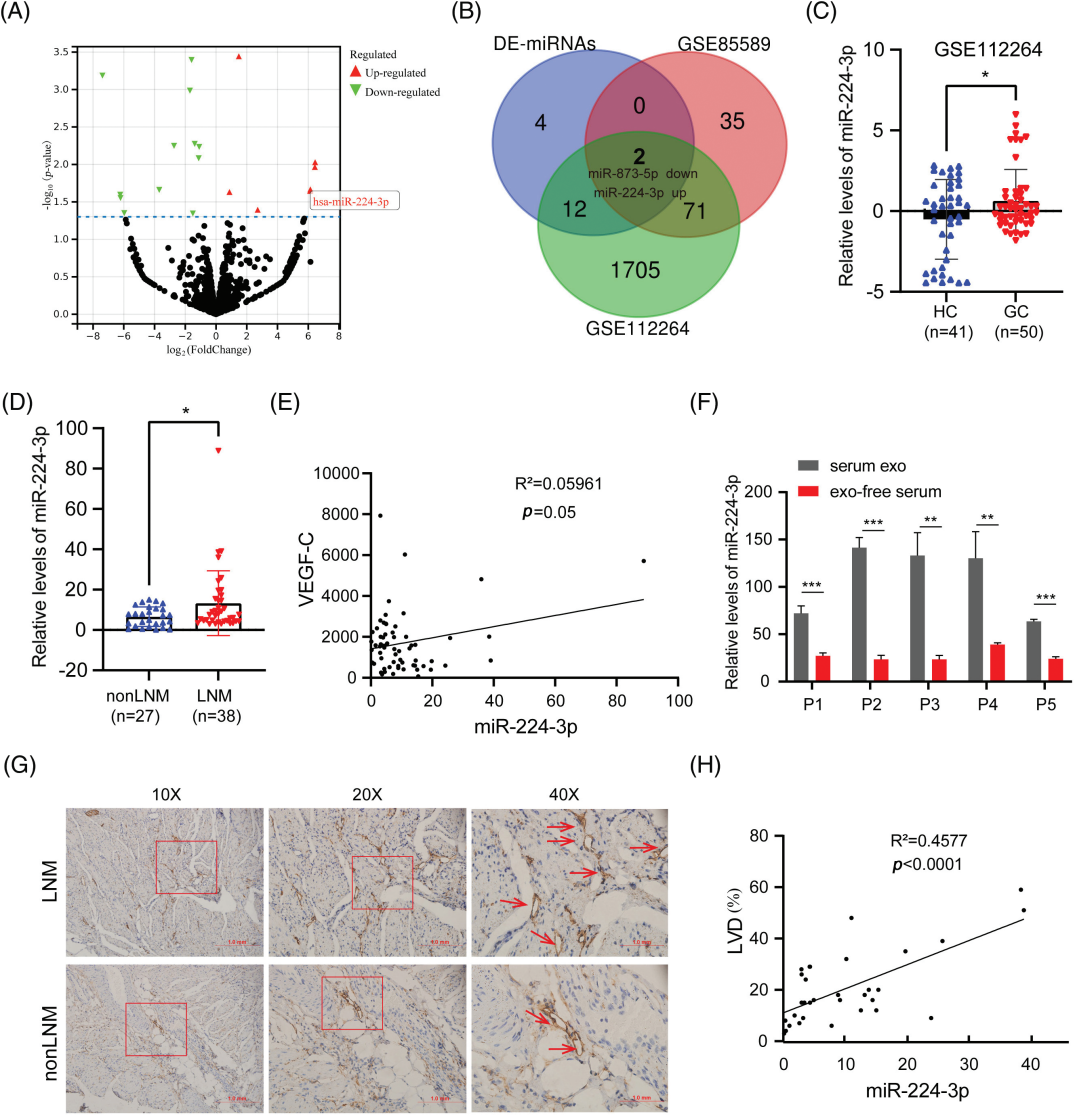
Exosomal miR-224 was screened to be associated with *LNM of GC* (A) Volcano plot analysis of serum sequencing differential miRNAs (LNM *vs*. nonLNM). (B) Venn diagram of the serum sequencing differential dataset *vs*. the GEO dataset. (C) Relative expression of serum miR-224-3p in HC and GC in GSE112264 datasets. (D) Relative expression of serum miR-224-3p in nonLNM and LNM. (E) Correlation analysis between the relative expression of serum miR-224-3p and serum VEGFC. (F) Relative expression of miR-224-3p in serum EVs and exo-free serum. (G) Schematic diagram of LVD in tumor tissues. (H) Correlation analysis between the relative expression of serum miR-224-3p and LVD in tumor tissues. *p-value < 0.05, **p-value < 0.01, ***p-value < 0.001.

Two noncoding RNA serum expression profiles were selected and screened from the GEO database: GSE112264 and GSE85589. The expression profile data and clinical information of the two datasets were downloaded. GC serum and healthy control (HC) serum were screened. The GSE112264 datasets contained 50 GC serum and 41 HC serum samples, while the GSE85589 datasets contained 7 GC serum and 19 HC serum samples. Differential analysis was performed between GC serum and HC serum extracted from GSE112264 and GSE85589, respectively. In GSE112264, 1790 differential serum miRNAs were screened, and 108 differential serum miRNAs were screened in GSE85589. In addition, 18 human-derived differential serum miRNAs (DE-miRNAs) were screened from the serum miRNA dataset analyzed by sequencing (LNM *vs*. nonLNM). MiR-224-3p was found in Fig. 1A. The Venn diagram of the three datasets was intersected to obtain an upregulated miRNA (miR-224-3p) and a downregulated miRNA (miR-873-5p) related to the LNM of GC (Fig. 1B). In this study, we focused on miR-224-3p.

**Figure A1:**
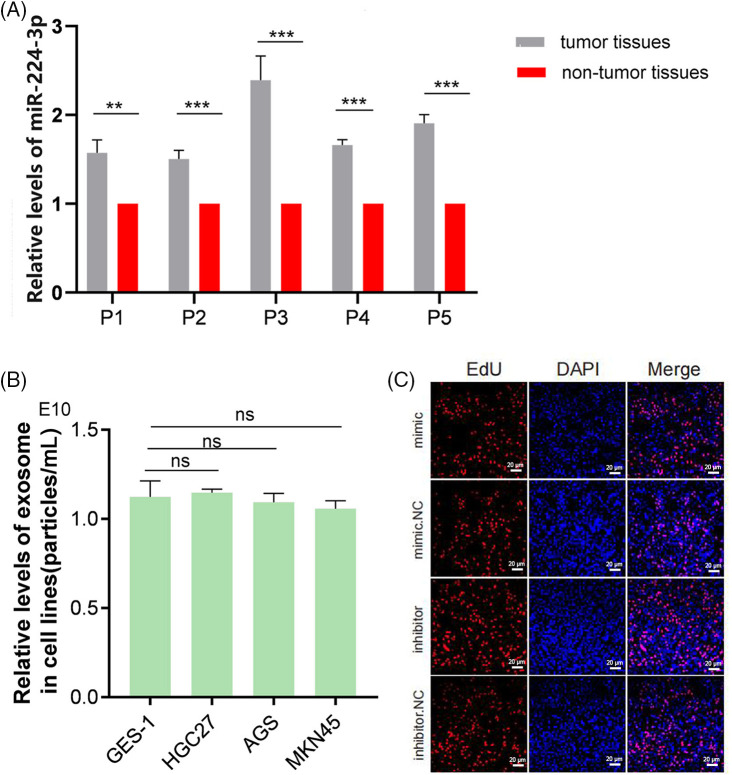
(A) Relative expression of miR-224-3p in tumor tissues and nontumor tissues. (B) Analysis of the levels of secreted EVs in all cell media. (C) No significant regulatory effect on the growth of GC cells at the cellular level by miR-224-3p. **p-value < 0.01, ***p-value < 0.001.

We extracted and analyzed the serum expression profile data of miR-224-3p. Compared with that in the HC group, the relative expression of serum miR-224-3p in the GC group was increased, suggesting that miR-224-3p may be involved in GC progression (Fig. 1C). We examined miR-224-3p expression in GC tissues, and the expression level in tumor tissues was significantly higher than that in nontumor tissues (Fig. A1(A)). Then, RNA was extracted from the collected serum of 65 patients with newly diagnosed GC, and the relative expression of miR-224-3p was measured by quantitative real-time PCR (qRT‒PCR). The expression of miR-224-3p in the serum of LNM patients (n = 38) was higher than that of nonLNM patients (n = 27), which demonstrated that serum miR-224-3p was probably associated with LNM in GC (Fig. 1D).

VEGFC is the most important cytokine that promotes lymphangiogenesis. Therefore, we detected the content of serum VEGFC in GC and explored the correlation between miR-224-3p and VEGFC in serum. There was almost no correlation between serum miR-224-3p and serum VEGFC (Fig. 1E). RNA was extracted from the serum EVs of patients and the serum after ultracentrifugation and measured the relative expression of miR-224-3p in each component. The relative expression level of exo-miR-224-3p in the serum of patients was significantly higher than that in the serum, which excluded EVs by ultracentrifugation (Fig. 1F). The results suggested that miR-224-3p in serum mainly existed in EVs.

To further explore the relationship between serum miR-224-3p and LNM in GC, tumor tissues from 32 GC patients were collected for immunohistochemistry (IHC), and the lymphatic endothelial marker padoplanin was used to evaluate the lymphatic vessel density (LVD) of tumor tissues. The amount of LVD in the LNM group was significantly higher than that in the nonLNM group (Fig. 1G). There was a positive correlation between GC serum miR-224-3p and LVD (Fig. 1H). The results indicated that miR-224-3p was associated with LNM in GC, while it may regulate LNM in GC in a VEGFC-independent manner.

### EVs derived from GC cells could deliver miR-224-3p into HLECs

**Figure 2:**
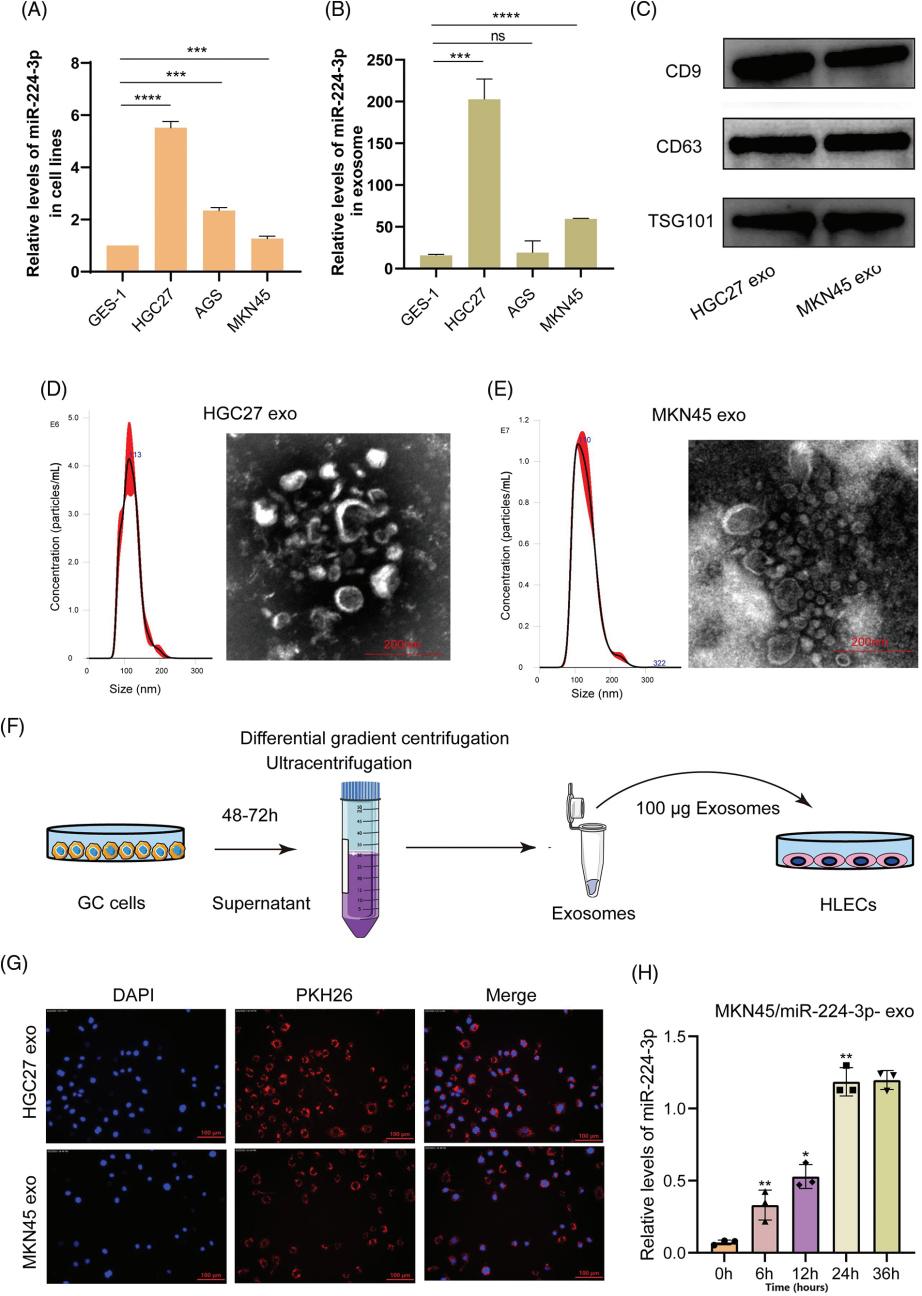
EVs derived from GC cells could deliver miR-224-3p into HLECs. (A) Analysis of the expression of miR-224-3p in GC-derived cell lines. (B) Analysis of the expression of miR-224-3p in EVs derived from GC cell lines. (C) Immunoblot analysis of exosomal signature proteins. (D and E) Electron microscopy and NTA analysis of (D) HGC27 and (E) MKN45 cell-derived EVs. (F) Diagram of coculture patterns for EV extraction. (G) EVs derived from GC cells PKH26 stained into HLECs. (H) The expression level of miR-224-3p was measured after HLECs were cocultured with EVs derived from MKN45 cells transfected with miR-224-3p mimic. *p-value < 0.05, **p-value < 0.01, ***p-value < 0.001, ****p-value < 0.0001.

The expression of miR-224-3p was increased in the serum of GC patients with LNM. Then, we investigated the expression of miR-224-3p in GC cell lines and their EVs. Compared with that in the normal gastric mucosal epithelial cell line GES-1, the expression of miR-224-3p was increased in the GC cell lines HGC27, AGS, and MKN45 (Fig. 2A). Meanwhile, we extracted EVs from each cell culture medium to measure the expression of miR-224-3p. The trend of exosomal miR-224-3p expression level secreted by each cell line was roughly the same as that secreted by cell lines (Fig. 2B). However, the levels of secreted EVs in all cell media were almost the same (Fig. A1(B)). The expression of miR-224-3p was the highest in HGC27 cells, and the expression of exo-miR-224-3p was also the highest in the culture medium. The expression of miR-224-3p in MKN45 cells and their EVs was also higher than that in GES-1 cells. Therefore, HGC27 and MKN45 cells were selected as EV donors in the subsequent study.

We collected HGC27 and MKN45 medium excluding serum EVs and then used differential gradient centrifugation to obtain a batch of purified HGC27/MKN45-derived particles. To prove that these extracted particles were EVs, we detected the characteristic proteins CD9, CD63, and TSG101 expressed by EVs. We found that three proteins are highly expressed in the particles obtained by this method (Fig. 2C). Under the electron microscope, these particles appeared as round or oval discs with a diameter of approximately 100 nm (Fig. 2D,E). The size of HGC-derived particles was 112.7 ± 25.8 nm, and the maximum size distribution was approximately 113 nm. The size of MKN45-derived particles was 109.2 ± 32.3.8 nm, and the maximum size distribution was approximately 110 nm. These results demonstrated that we successfully obtained purified EVs for subsequent experiments.

EVs obtained by ultracentrifugation were labeled with the fluorescent dye PKH26 and then cocultured with human lymphatic endothelial cells (HLECs), as shown in Fig. 2F. After 4–6 h, we observed a large number of cocultured EVs endocytosed by HLECs or bound around HLECs under a fluorescence microscope (Fig. 2G). In addition, we extracted RNA from HLECs cocultured with EVs after 0, 6, 12, 24, and 36 h and measured the expression level of miR-224-3p. It was revealed that the relative expression of miR-224-3p increased gradually with the prolongation of coculture time (Fig. 2H).

### Exo-miR-224-3p from GC cells promoted the tube formation and migration of HLECs

**Figure 3:**
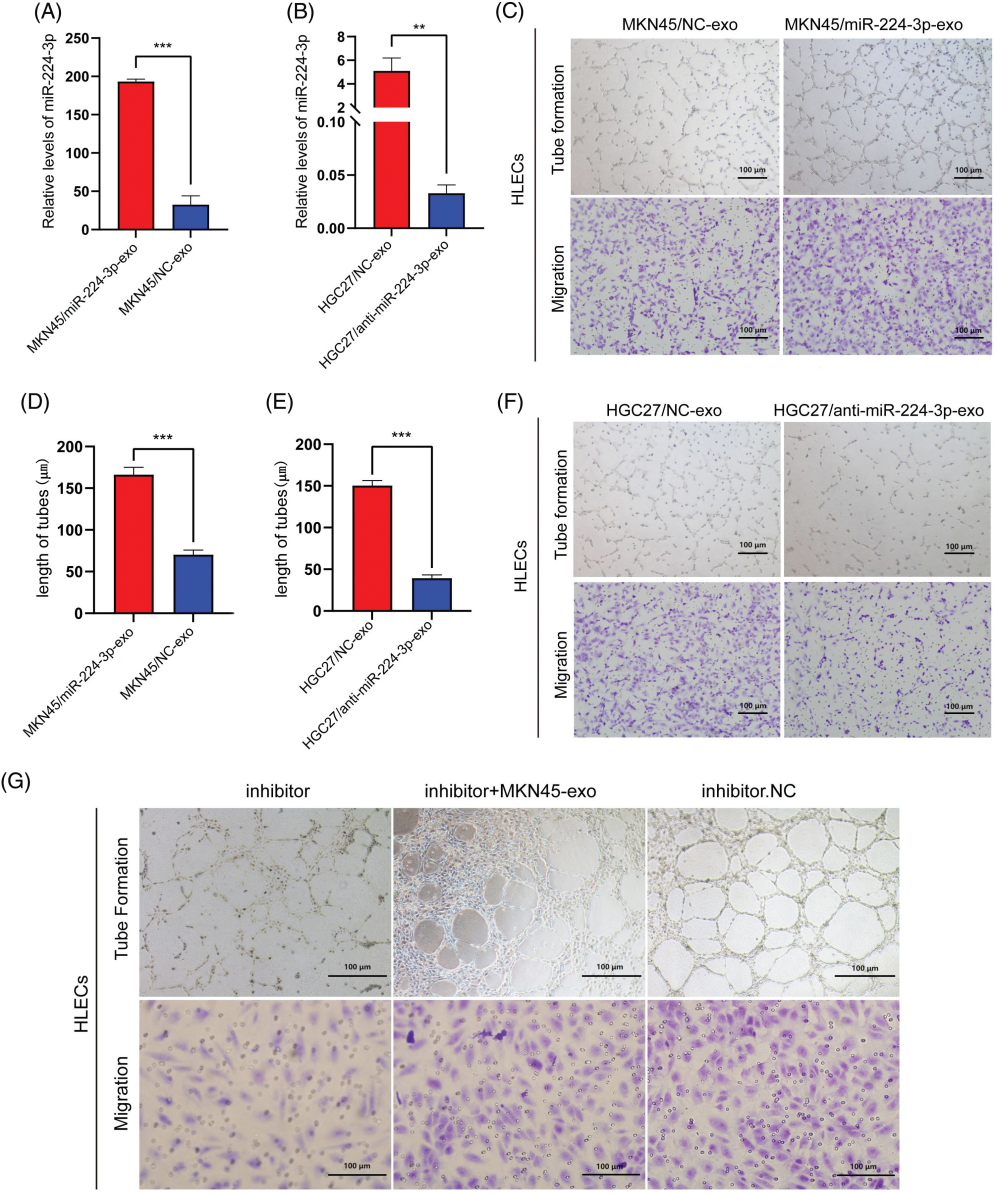
Exo-miR-224-3p from GC cells promoted the tube formation and migration of HLECs (A) The expression level of miR-224-3p in EVs from MKN45 cells. (B) The expression level of miR-224-3p in EVs from HGC27 cells. (C) Effects of exosomal miR-224-3p from differently transfected MKN45-derived EVs on the tube formation and migration of HLEC cells. (D) Length of tubes from differently transfected MKN45-derived EVs. (E) Length of tubes from differently transfected HGC27-derived EVs.Effects of exosomal miR-224-3p from differently transfected HGC27-derived EVs on the tube formation and migration of HLEC cells. (F) Effects of exosomal miR-224-3p from differently transfected HGC27-derived EVs on the tube formation and migration of HLEC cells. (G) Changes in the tube formation and migration ability of differently transfected MKN45 cells in the rescue experiment. *p-value < 0.05, **p-value < 0.01, ***p-value < 0.001.

MKN45 and HGC27 cells were transfected with miR-224-3p mimic (MKN45/miR-224-3p-exo) or miR-224-3p inhibitor (HGC27/anti-miR-224-3p-exo) and the corresponding normal control (NC) and were cultured with medium excluding EVs for 48–72 h. EVs were extracted from the culture medium to determine the relative expression of miR-224-3p. Compared with that in the NC group, the expression level of miR-224-3p in the MKN45/miR-224-3p-exo group was significantly increased (Fig. 3A). However, the expression level of miR-224-3p was observably decreased in the HGC27/anti-miR-224-3p-exo group (Fig. 3B). The results indicated that EVs derived from GC could function as mediators to regulate the expression level of miR-224-3p in HLECs and facilitate communication between HLECs and GC cells in the microenvironment.

Next, we explored the effect of exo-miR-224-3p on the biological behavior of HLECs. The number of lymphatic branch tubes formed by HLECs in matric gel represented the lymphangiogenesis ability, and the Transwell assay was used to detect the change in the migration ability. Compared with those in the NC group, HLECs in the MKN45/miR-224-3p-exo group had significantly enhanced lymphangiogenesis ability and migration ability (Fig. 3C,D). However, these abilities of HLECs in the HGC27/anti-miR-224-3p-exo group were impaired (Fig. 3E,F). To further confirm that the stronger lymphangiogenic ability of HLECs is caused by the change in miR-224-3p expression in EVs, we performed a rescue experiment (Fig. 3G). The results proved that exo-miR-224-3p indeed affected the biological functions of HLECs.

### Exo-miR-224-3p derived from GC cells could promote LNM in vivo

MKN45 cells were infected with lentivirus overexpressing luciferase. The aminoglycoside antibiotic G418 was used in the drug screening test for the stable line with high expression of luciferase (MKN45-Luci). Then, MKN45-Luci cells were infected with lentivirus overexpressing miR-224-3p or sponge miR-224-3p and the corresponding NC. Finally, these cells were screened by puromycin to obtain MKN45-Luci-OE, MKN45-Luci-SP, and MKN45-Luci-NC cells.

**Figure 4:**
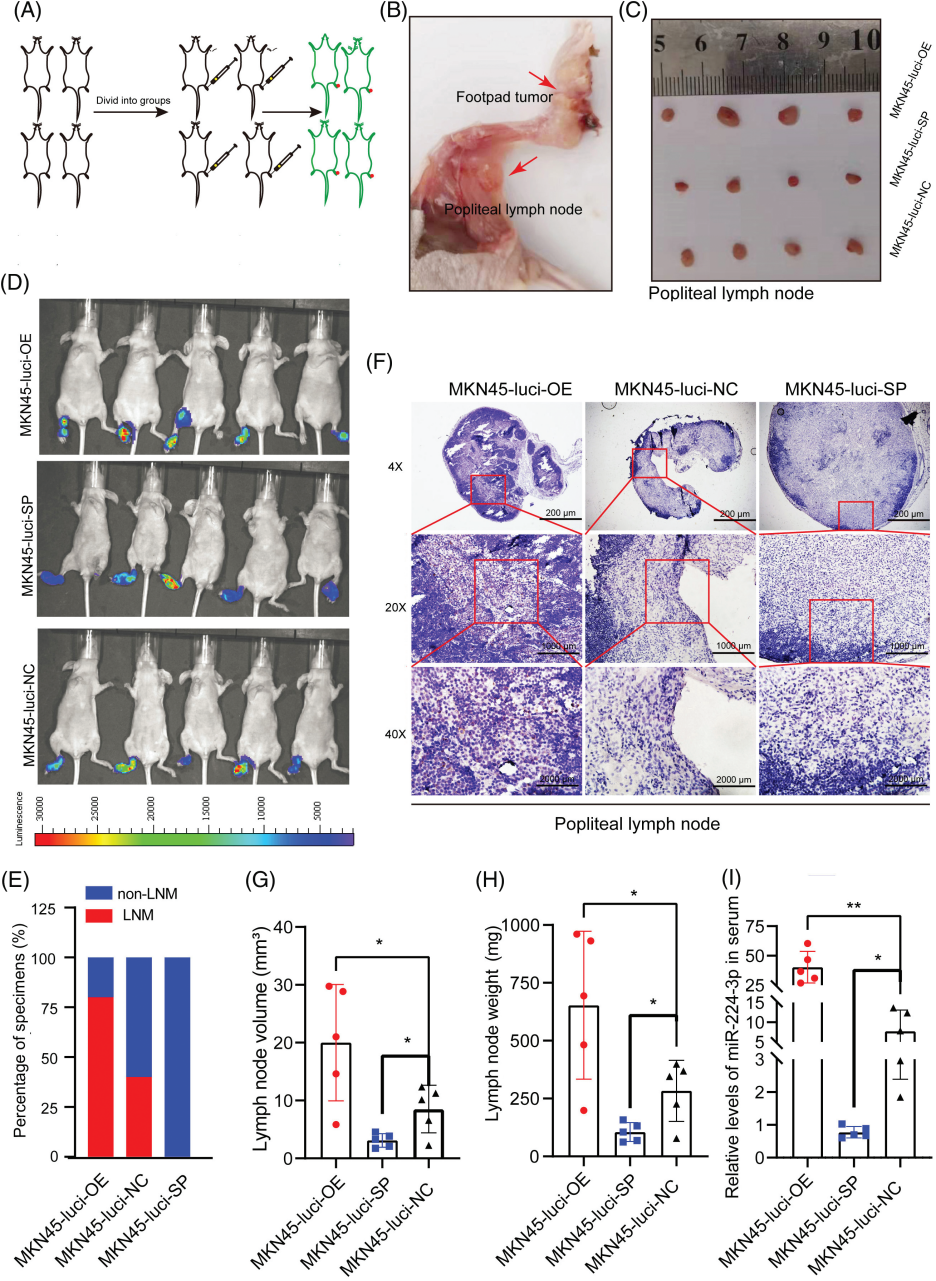
Exo-miR-224-3p derived from GC cells could promote LNM *in vivo* (A) Flow chart of the construction of the mouse foot pads LNM model. (B) Schematic representation of popliteal metastatic lymph node dissection in foot pad xenograft tumors. (C) Schematic representation of popliteal lymph node size in each group. (D) LNM was analyzed by imaging small animals *in vivo*. (E) Analysis of the LNM rate by imaging small animals *in vivo*. (F) Analysis of the expression of luciferase in the popliteal lymph nodes of nude mice in each group by IHC. (G) The volume of popliteal lymph nodes in each group (n = 5). (H) Weights of popliteal lymph nodes in each group (n = 5). (I) The expression of serum miR-224-3p in each group of nude mice (n = 5). *p-value < 0.05, **p-value < 0.01.

The animal experiments are carried out in accordance with the described part in the experimental methods (Fig. 4A–C). The results of *in vivo* imaging showed that four nude mice in the MKN45-Luci-OE group developed popliteal LNM, with a metastasis rate of 80%. No metastases were found in the MKN45-Luci-SP group, whereas two metastases occurred in the MKN45-Luci-NC group (Fig. 4D,E). To more accurately observe the LNM status of xenografts, a luciferase antibody was used to immunostain the popliteal lymph nodes. Compared with that of the MKN45-Luci-NC group, the MKN45-Luci-OE group had a higher positive rate of luciferase cells, while the MKN45-Luci-SP group had almost no luciferase expression (Fig. 4F). The volume and weight of lymph nodes in the three groups are shown in Fig. 4G,H. The expression of miR-224-3p was measured in the serum of nude mice in each group. Serum from each group of mice was collected and the expression level of miR-224-3p was detected. The expression of serum miR-224-3p in the MKN45-Luci-OE group was higher than that in the MKN45-Luci-NC group, and the expression of serum miR-224-3p in the MKN45-Luci-SP group was lower than that in the MKN45-Luci-NC group (Fig. 4I). It was demonstrated that overexpression of miR-224-3p could promote LNM of GC *in vivo*.

### Exo-miR-224-3p derived from GC cells could promote lymphangiogenesis in vivo

**Figure 5:**
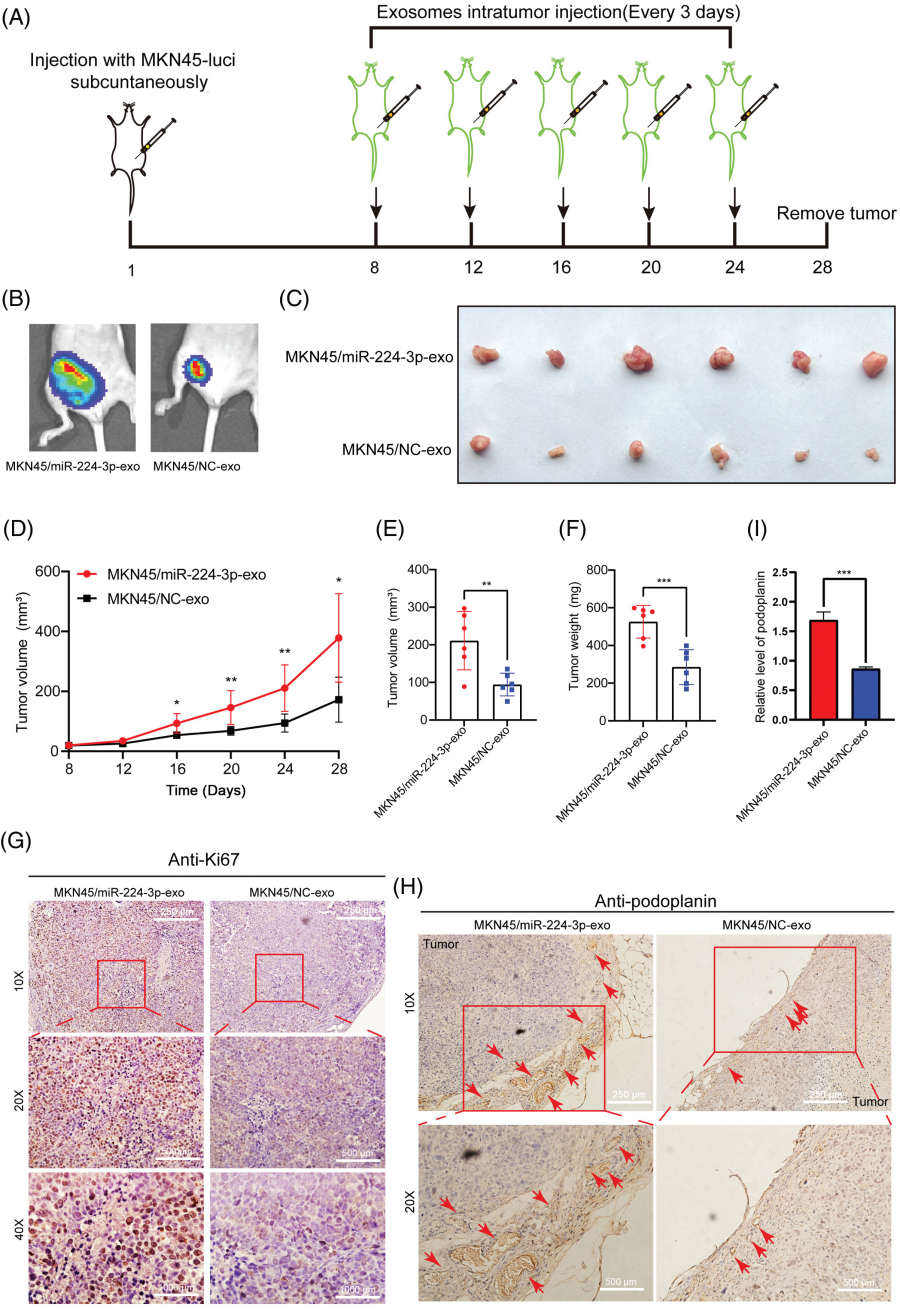
Exo-miR-224-3p derived from GC cells could promote lymphangiogenesis *in vivo*. (A) Flow chart of the construction of a subcutaneous xenograft tumor model in mice. (B) Analysis of subcutaneous xenografts by imaging of small animals *in vivo*. (C) Schematic diagram of transplanted tumors in each group (n = 6). (D) Measurement of transplanted tumor volume in nude mice of each treatment group (n = 6). (E) Volume of transplanted tumors in nude mice of each treatment group on Day 28 (n = 6). (F) Weight measurement of transplanted tumors in nude mice of each treatment group on Day 28 (n = 6). (G) The expression of Ki67 in xenograft tumor tissues was analyzed by IHC. (H) The expression of podoplanin in xenograft tumor tissues was analyzed by IHC. (I) Relative expression level of podoplanin in xenograft tumor tissues was analyzed by qRT-PCR. *p-value < 0.05, **p-value < 0.01, ***p-value < 0.001.

To explore the effect of miR-224-3p on lymphangiogenesis *in vivo*, the constructed MKN45-Luci cells were implanted subcutaneously in nude mice (Fig. 5A). The animal experiments are carried out in accordance with the described part in the experimental methods. We can see that the fluorescence area and intensity of the MKN45/miR-224-3p group were larger than those of the MKN45/NC-exo group (Fig. 5B). After that, the nude mice were sacrificed, and the transplanted tumors were removed (Fig. 5C). The curve of tumor growth volume change suggested that the MKN45/miR-224-3p-exo group had faster tumor growth and larger tumor volume (Fig. 5D). The volume and weight of tumor tissues in each group are shown in Fig. 5E,F. Ki67 antibody, a tumor cell proliferation marker, was used to detect the proliferation of tumor cells in the transplanted tumor tissues by IHC [26]. It was found that the proliferation of tumor cells in the MKN45/miR-224-3p-exo group became vigorous, which was consistent with the trend of tumor volume and weight (Fig. 5G). Furthermore, to verify the specific effects of exo-miR-224-3p on lymphangiogenesis in GC, podoplanin antibody, which is a specific marker of HLECs that can reveal the status of lymphangiogenesis in and around tumors by IHC, was used to perform IHC on the removed tumor tissues. The results indicated that the density of lymphatic vessels around tumor tissues was higher in the MKN45/miR-224-3p-exo group than in the MKN45/NC-exo group (Fig. 5H,I). Importantly, miR-224-3p had no significant regulatory effect on the growth of GC cells at the cellular level (Fig. A1(C)). In conclusion, it was confirmed that exo-miR-224-3p could promote tumor lymphangiogenesis *in vivo*.

### Exo-miR-224-3p targeted GSK3B to promote lymphangiogenesis

**Figure 6:**
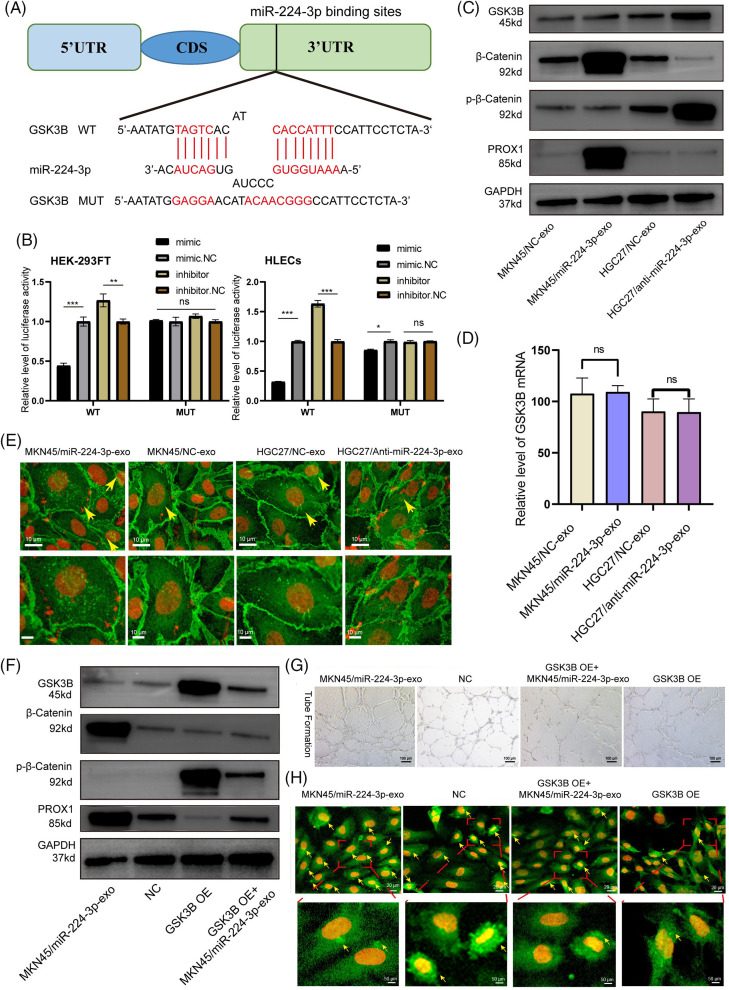
Exo-miR-224-3p targeted GSK3B to promote lymphangiogenesis. (A) Predicted pattern of binding sites between miR-224-3p and the GSK3B mRNA 3’UTR. (B) The binding sites between miR-224-3p and the 3’UTR of GSK3B mRNA were analyzed by dual luciferase reporter assay in the HEK-293FT cell line and HLEC cell line. (C) Analysis of the effect of exosomal miR-224-3p expression changes on GSK3B protein levels, β-catenin levels, phosphorylation levels, and PROX1 protein expression levels by WB. (D) Analysis of the effect of changes in the expression of exosomal miR-224-3p on GSK3B mRNA by qRT-PCR. (E) The expression and localization of β-catenin in HLECs treated with different EVs under a fluorescence microscope. Red: DAPI; Green: β-catenin. The scale bar is 10 μm. (F) Detection of the protein expression of different treatment groups by WB. (G) Effect of restoring GSK3B protein expression with the GSK3B overexpression plasmid on the tube formation ability of HLECs. (H) Analysis of the expression and localization of β-catenin in HLECs in different treatment groups. Red: DAPI. Green: β-catenin. The scale bar is 20 μm. *p-value < 0.05, **p-value < 0.01, and ***p-value < 0.001.

Online database miWalk (http://mirwalk.umm.uni-heidelberg.de/, accessed on 01/05/2024), DIANA (http://diana.imis.athenainnovation.gr/DianaTools/index.php?r=microT_CDS/index, accessed on 01/05/2024) and TargetScan (http://www.targetscan.org/vert_72/, accessed on 01/05/2024) was combined to explore miR-224-3p potential targets. A binding site between miR-224-3p and the 3′UTR of GSK3B was found. HEK293FT and HLECs were used to verify the targeting regulatory relationship between miR-224-3p and GSK3B by the dual luciferase reporter assay. There were two groups, wild-type (WT) and mutant type (MUT), and the mutant-type group contained mutated plasmids targeting the binding site. The binding site of miR-224-3p and the GSK3B mRNA 3′UTR predicted is shown in Fig. 6A. In HEK293FT and HLECs, the fluorescence value of the WT group transfected with miR-224-3p mimic was significantly decreased compared with that of the NC group, while that of the WT group transfected with miR-224-3p inhibitor was increased. However, there was no significant difference in the fluorescence value of the MUT group, whether transfected with miR-224-3p mimic or miR-224-3p inhibitor (Fig. 6B).

We continued to explore the specific regulatory relationship between miR-224-3p and GSK3B. EVs derived from MKN45/miR-224-3p-exo, MKN45/NC-exo, HGC27/anti-miR-224-3p-exo and HGC27/NC-exo was collected. Then, they were cocultured with HLECs. The expression of GSK3B was detected by Western blot (WB). The protein level of GSK3B in the MKN45/miR-224-3p-exo group was significantly decreased compared with that in the MKN45/NC-exo group, while the protein level of GSK3B in the HGC27/anti-miR-224-3p-exo group was higher than that in the HGC27/NC-exo group (Fig. 6C). No significant difference was found in GSK3B mRNA levels among these groups by qRT-PCR (Fig. 6D). The results suggested that the GSK3B protein level may be regulated posttranscription, and the expression of GSK3B in HLECs could be suppressed by exo-miR-224-3p derived from GC.

We further investigated the possible mechanism by which altered GSK3B expression affects lymphangiogenesis. In the classical Wnt-β-catenin signaling pathway, β-catenin in the cytoplasm is phosphorylated and degraded by GSK3B. The inhibition of the GSK3B protein expression leads to the accumulation of β-catenin in the cytoplasm and the activation of β-catenin that is transported into the nucleus as a transcription factor to promote gene transcription [23,24]. It was reported that the entry of β-catenin into the nucleus could enhance the expression of Prospero homeobox 1 (PROX1), thereby promoting lymphangiogenesis [25]. Next, we aimed to verify whether the change in the miR-224-3p expression level would affect the stable entry of β-catenin into the nucleus and promote the expression of PROX1 after affecting the expression of GSK3B. Inhibiting the expression of GSK3B decreased the phosphorylation level of β-catenin. The degradation of β-catenin was reduced leading to its β-catenin stabilization (Fig. 6C).

To verify whether β-catenin aggregates in the cytoplasm and increases in nuclear entry, MKN45/miR-224-3p-exo, MKN45/NC-exo group, HGC27/anti-miR-224-3p-exo and HGC27/NC-exo was used for immunofluorescence to detect the expression and distribution of β-catenin. After coculture with MKN45/miR-224-3p-exo and HLECs, β-catenin accumulated in the cytoplasm and increased in the nucleus of HLECs compared with those in the MKN45/NC-exo group. However, compared with those in the HGC27/NC-exo group, the accumulation and nuclear localization of β-catenin in HLECs in the HGC27/anti-miR-224-3p-exo group were decreased (Fig. 6E). Moreover, the expression of PROX1 in the lysates of each group was measured. Compared with that in the MKN45/NC-exo group, the expression level of PROX1 in the MKN45/miR-224-3p-exo group was upregulated, while in the HGC27/anti-miR-224-3p-exo group was downregulated.

To clarify that GSK3B is the direct target of exo-miR-224-3p affecting HLECs, a GSK3B overexpression plasmid was transfected into HLECs (GSK3B OE). Then, they were cocultured with EVs derived from GC cells transfected with miR-224-3p mimic (GSK3B OE + MKN45/miR-224-3p-exo). After overexpression of GSK3B, the protein level of p-β-catenin was significantly increased, while that of β-catenin was slightly decreased, indicating that GSK3B promoted the phosphorylation and degradation of β-catenin by inhibiting the β-catenin signaling pathway (Fig. 6F). However, compared with that in the GSK3B OE group, the expression level of p-β-catenin was partially suppressed, and β-catenin expression was slightly increased in the GSK3B OE+ MKN45/miR-224-3p-exo group. The tube formation ability of HLECs in the GSK3B group was significantly decreased compared with that in the NC group (Fig. 6G). While it was observed that the tube formation ability of HLECs in the MKN45/miR-224-3p-exo group was somewhat enhanced, this ability was still worse than that in the NC group. In addition, immunofluorescence experiments were performed on the above treatment groups (Fig. 6H). The intracellular nuclear localization of β-catenin in the GSK3B OE group decreased significantly. The intracellular nuclear localization of β-catenin in the GSK3B OE + MKN45/miR-224-3p-exo group was increased compared with that in the GSK3B OE group. We also found that the expression level of PROX1 decreased in the GSK3B OE group and increased slightly in the GSK3B OE + MKN45/miR-224-3p-exo group. In conclusion, exo-miR-224-3p derived from GC directly targets GSK3B in HLECs, inhibits the phosphorylation and degradation of β-catenin, and promotes the nuclear activation of β-catenin, leading to the transcription of PROX1 and thus affecting lymphangiogenesis.

### hnRNPA1 mediated the sorting of miR-224-3p into EVs

**Figure 7:**
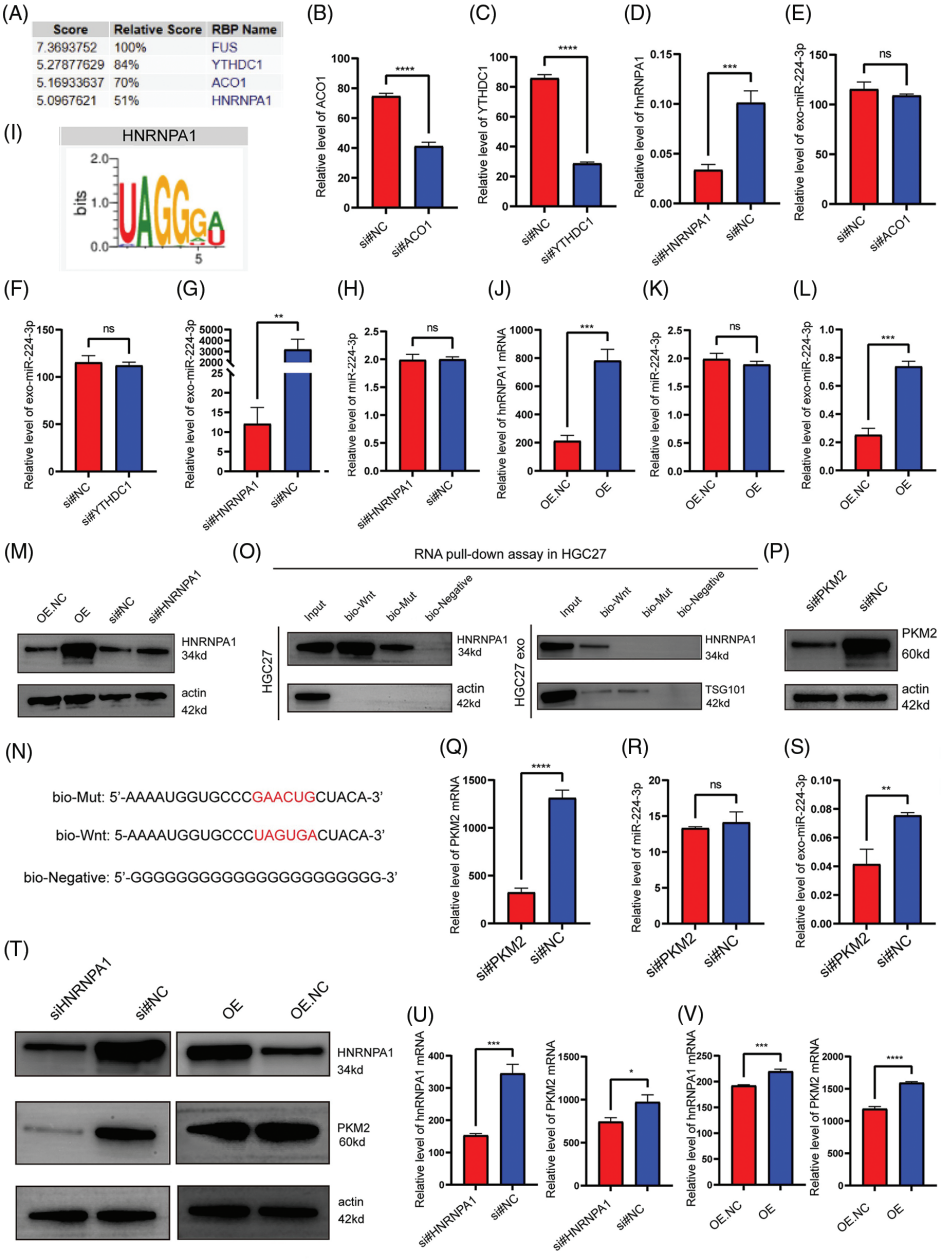
hnRNPA1 mediated the sorting of miR-224-3p into EVs. (A) The RBPDB database was used to predict and analyse the RBPs that had binding motifs with miR-224-3p. (B–D) Analysis of (B) ACO1, (C) YTHDC1, and (D) hnRNPA1 expression in HGC27 cells after transfection with siRNA for knockdown by qRT‒PCR. (E–G) Analysis of the expression of exosomal miR-224-3p in HGC27 cells after knockdown of (E) ACO1, (F) YTHDC1, and (G) hnRNPA1 with siRNA by qRT‒PCR. (H) Analysis of the expression of miR-224-3p in HGC27 cells after knockdown of hnRNPA1 with siRNA by qRT‒PCR. (I) The motif of hnRNPA1 was predicted to bind miR-224-3p by the RBPDB database. (J) Analysis of the expression of hnRNPA1 mRNA in HGC27 cells after overexpression of hnRNPA1 by the plasmid. (K) Analysis of the expression of miR-224-3p in HGC27 cells after knockdown of hnRNPA1 by siRNA. (L) Analysis of the expression of exosomal miR-224-3p in HGC27 cells after overexpression of hnRNPA1 plasmid. (M) Analysis of plasmid overexpression and knockdown of hnRNPA1 in HGC27 cells by WB. (N) Biotin-tagged sequences were used for miRNA pull-down analysis. (O) Western blotting analysis of hnRNPA1 expression in HGC27 cell lysates or EV lysates and miRNA pull-down samples labelled with biotinylated miR-196a sequence (Bio-Wnt) or mutated miR-196a sequence (Bio-Mut). Biotinylated poly (G) (Bio-Negative) was used as a negative control. (P) Analysis of siRNA transfection to knockdown PKM2 in HGC27 cells by WB. (Q) Analysis of transfected siRNA knockdown of PKM2 in HGC27 cells. (R) Analysis of the expression of miR-224-3p in HGC27 cells after PKM2 knockdown by siRNA. (S) Analysis of the expression of exosomal miR-224-3p in HGC27 cells after transfection of siRNA to knock down PKM2. (T) The protein expression of PKM2 in HGC27 cells after different transfections by WB. (U) Analysis of the mRNA expression of hnRNPA1 and PKM2 in HGC27 cells after transfection of siRNA to knock down hnRNPA1. (V) Analysis of the mRNA expression of hnRNPA1 and PKM2 in HGC27 cells after transfection of a plasmid overexpressing hnRNPA1. *p-value < 0.05, **p-value < 0.01, ***p-value < 0.001, ****p-value < 0.0001.

To investigate how miR-224-3p is specifically sorted into EVs in GC cells, we analyzed the specific interactions between miR-224-3p sequences and RNA-binding protein (RBP) motifs via the RBPDB database (database of RBP specificities, http://rbpdb.ccbr, accessed on 01/05/2024). The analysis showed that there were three RBPS with scores greater than 5 and binding bases greater than 4 to miR-224-3p (Fig. 7A). These RBPS are YTH Domain Containing 1 (YTHDC1), Aconitase 1 (ACO1), and heterogeneous nuclear ribonucleoprotein A1 (hnRNPA1). Specific siRNAs were designed for YTHDC1, ACO1, and hnRNPA1. These siRNAs effectively reduced the expression levels of YTHDC1, ACO1, and hnRNPA1 (Fig. 7B–D). The expression level of miR-224-3p in EVs transfected with YTHDC1 siRNA or ACO1 siRNA did not change significantly, while that of miR-224-3p in EVs transfected with hnRNPA1 siRNA decreased (Fig. 7E–G). These results suggested that hnRNPA1 could affect the expression level of exo-miR224-3p, but YTHDC1 and ACO1 could not.

It was hypothesized that hnRNPA1 might affect the abundance of exo-miR-224-3p by affecting the expression of total miR-224-3p in cells. To verify this hypothesis, HGC27 cells were transfected with hnRNPA1 siRNA. The knockdown of hnRNPA1 had no significant effect on the expression of miR-224-3p (Fig. 7H). In recent years, it was reported that the heterogeneous nuclear ribonucleoprotein (hnRNP) family interacts with miRNA motifs to mediate the sorting of miRNAs into EVs, such as hnRNPA2B1 [27]. Considering similar reports from the literature and our results, hnRNPA1 may be involved in the sorting of miR-224-3p into EVs. The motif of miR-224-3p binding to hnRNPA1 was predicted by the RPBDP database (Fig. 7I).

To further clarify that hnRNPA1 may participate in the sorting of miR-224-3p into EVs, a plasmid overexpressing hnRNPA1 was designed (Fig. 7J). HGC27 cells were transfected with the plasmid, and the expression levels of miR-224-3p in EVs and cells were measured by qRT‒PCR. It was found that after overexpression of hnRNPA1, the level of exosomal miR-224-3p increased, but the increase was not obvious in HGC27 cells (Fig. 7K,L). HnRNPA1 was effectively knocked down or overexpressed in HGC27 cells (Fig. 7M). Moreover, targeting the predicted binding site of hnRNPA1 and miR-224-3p (UAGUGA sequence), we synthesized miR-224-3p with biotin (bio-Wnt), mutated miRNA with biotin (bio-Mut) and bionegative control sequence with biotin (bio-Negative), as shown in Fig. 7N. It displays the results of miRNA pull-down (Fig. 7O). The interaction between hnRNPA1 and miR-224-3p was observed in whole cell lysates, and miR-224-3p could pull out hnRNPA1. However, when the UAGUGA sequence of miR-224-3p was mutated, its binding ability to hnRNPA1 was impaired. The enrichment of hnRNPA1 in the bio-Wnt group was also found in EV lysates. It was found that hnRNPA1 played a regulatory role in the level of exo-miR-224-3p and mediated the sorting of miR-224-3p into EVs.

Finally, we investigated the effect of PKM2 on the secretion of exo-miR-224-3p. HGC27 cells were transfected with siRNA to reduce the expression of PKM2 (Figs. 7P–S). Compared with that in the NC group, the exo-miR-224-3p from cell lines with downregulated expression of PKM2 was significantly reduced, while the miR-224-3p in the cell lines was not significantly changed, which suggested that PKM2 could promote the secretion of exo-miR-224-3p derived from GC cells. However, the specific mechanism by which PKM2 promotes secretion needs to be further explored. In addition, hnRNPA1, as a splicing protein, has been reported to splice intracellular PKM in the direction of PKM2. Therefore, we transfected the hnRNPA1-overexpressing plasmid or siRNA and corresponding NC into HGC27 cells. Overexpression of hnRNPA1 increased the mRNA and protein expression of PKM2, while knockdown of hnRNPA1 downregulated both the mRNA and protein expression of PKM2 (Fig. 7T–V). The results indicated that hnRNPA1 promoted the secretion of exo-miR-224-3p by enhancing the expression of PKM2, together with PKM2.

## Discussion

As one of the five most common tumors in the world, GC has a strong tendency towards LNM, and lymph node involvement is a factor closely related to prognosis [1,28]. With the increase in the number of metastatic lymph nodes, the survival rates of GC patients decrease significantly [29]. In addition, the overall survival of patients with nonLNM is longer than that of patients with LNM, and the overall recurrence rate is significantly lower than that of patients with LNM, which has become a clinical consensus [30,31]. Tumor EVs carry a large number of cancer-related molecular fingerprints, which can reflect the biological behavior of tumor cells [32,33]. It has been reported that LNM GC cells can induce bone marrow mesenchymal stem cells to enter the metastatic lymph node microenvironment through the activation of YAP signaling by exo-Wnt5a, which provides new insight into the mechanism of LNM in GC [34]. Therefore, strategies that block the sorting, transport, or uptake of EVs may be effective treatments or novel targets for GC.

The VEGF protein family consists of VEGFA, VEGFB, VEGFC, VEGFD, and many others, some members of which are inducers of tumor lymphangiogenesis [35,36]. VEGFC-VEGFCR3 and VEGFD-VEGFR3 signaling pathways are considered to be major drivers of tumor lymphangiogenesis and lymphatic remodeling [37,38]. The binding of VEGFC and VEGFD to VEGFR3 leads to receptor homodimerization and autophosphorylation and then activates downstream lymphangiogenic signals of the RAS-MAPK and PI3K-Akt pathways [38,39]. In addition to the VEGF family, fibroblast growth Factor 2 and platelet-derived growth Factor B also have lymphangiogenic activity [40,41]. Lymphangiogenesis in tumors involves a complex interaction of related stimulatory molecules, which regulate signaling pathways to induce lymphatic endothelial cell proliferation, sprouting, migration, and finally the formation of new networks from the existing lymphatic portion [38]. In our study, no correlation was found between the expression level of EV contents and serum VEGFC, suggesting that there may be a novel VEGFC-independent mechanism that promotes lymphangiogenesis and metastasis in GC.

PROX1 is a key transcription factor that drives the fate of HLECs by regulating the expression of various lymphatic vessel-specific proteins, including VEGFR3, LYVE-1, and podoplanin [42–44]. Overexpression of PROX1 can induce the proliferation and migration of HLECs, thereby promoting lymphangiogenesis. It has been reported that exosomal long noncoding RNA LNMAT2 upregulates PROX1 in HLECs to promote bladder cancer lymphangiogenesis and LNM [42]. As a pivotal factor in the classical WNT/β-catenin signaling pathway, abnormal activation of β-catenin plays an important role in the occurrence and progression of various human tumors [45–47]. Recent evidence indicates that activation of β-catenin signaling can accelerate the transcription of PROX1 to regulate the formation of lymph tubes [47]. The β-catenin and PROX1 complex may promote FOXC1 expression and regulate lymphatic vessel development [48]. In recent years, some studies have reported that GSK3B inhibition is involved in the regulation of lymphangiogenesis through a β-catenin-dependent pathway [25,49]. On the other hand, GSK3B inhibition can activate the mTOR pathway [25,50,51]. Therefore, GSK3 inhibition can regulate the lymphangiogenic developmental pathway by stabilizing β-catenin or by activating mTOR signaling. In this study, we demonstrate that the inhibition of GSK3B expression reduces β-catenin phosphorylation and promotes PROX1 transcription, leading to tumor lymphangiogenesis.

Researchers have found that the entry of miRNA into EVs is not a random process [52]. Some intracellular miRNAs are specifically selected into EVs through a specific sorting process [53]. RBPs such as hnRNPC1, hnRNPK, and hnRNPA1 are involved in exo-miRNA transport by binding to specific motifs [27,54]. Previous studies in our research group showed that hnRNPA1 participated in the sorting of miR-522 into fibroblast EVs, which inhibited ferroptosis and obtained chemotherapeutic resistance in GC [22]. Furthermore, it has been reported that PKM2 also plays a role in the secretion of EVs [18,55]. Wu et al. found that sumoylated PKM2 in liver cancer cells could interact with arrestin dome-containing protein 1, which is localized at the plasma membrane to mediate microvesicle budding and exocytosis in the form of microvesicles [18]. In this study, we explored the effect of PKM2 expression levels in GC cells on the secretion of exo-miR-224-3p. The preliminary conclusion is that the expression of PKM2 can promote the secretion of exo-miR-224-3p, but the specific mechanism needs to be elucidated in future studies.

**Figure 8:**
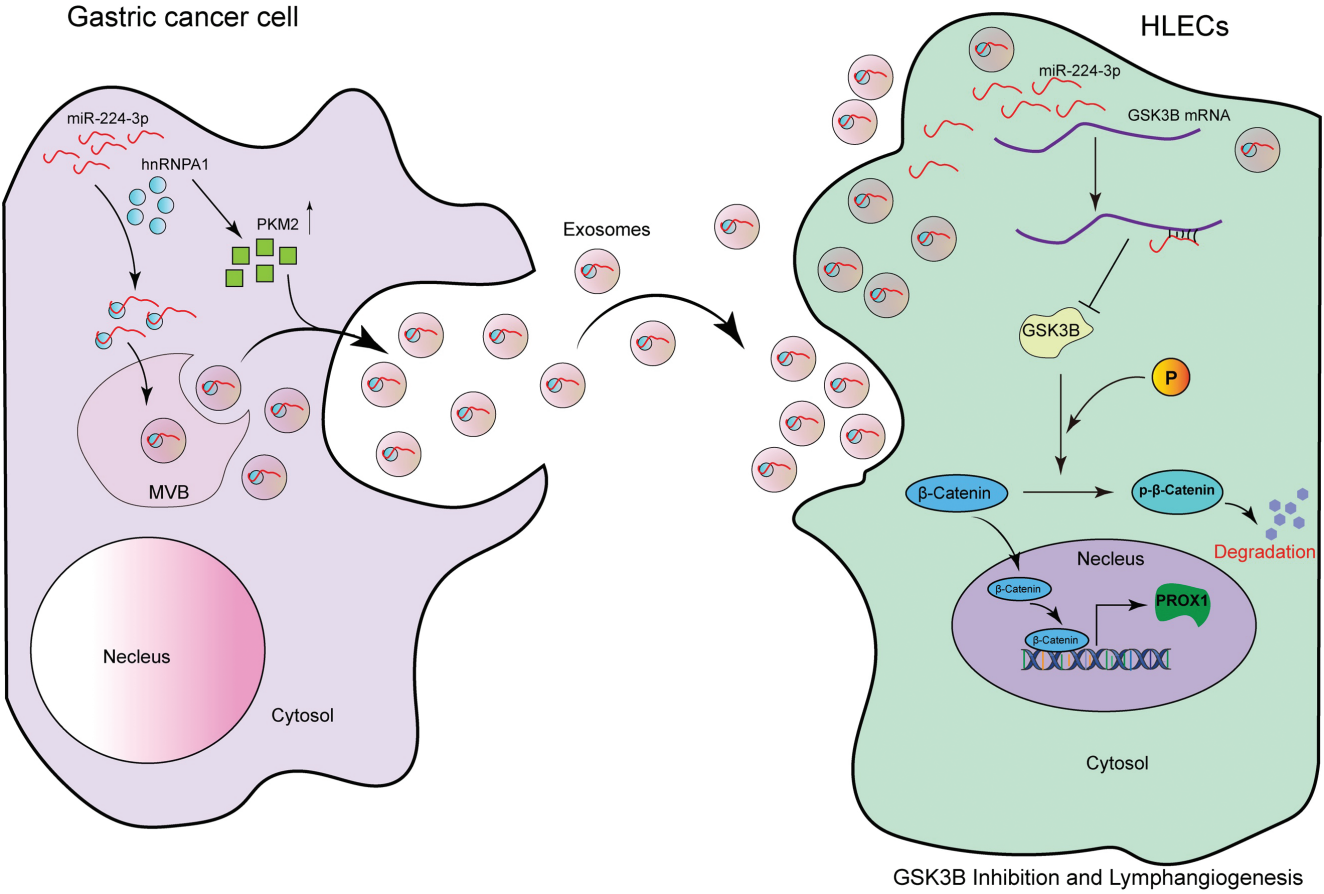
The model of the GC cell-secreted exo-miR-224-3p-mediated GSK3B/β-catenin/PROX1 axis in HLECs for promoting lymphangiogenesis and LNM.

In this study, we found that exo-miR-224-3p derived from GC cells could be taken up by HLECs and promote their formation and migration (Fig. 8). The regulatory axis of GC cells-exosomal miR-224-3p-HLECs-GSK3B/β-catenin-PROX1 was demonstrated for the first time, providing a theoretical basis for exo-miR-224-3p as an intervention target for lymphangiogenesis and LNM in GC. In addition, we elucidated the mechanism by which miR-224-3p was sorted into EVs by hnRNPA1 in GC cells, and PKM2 could promote the secretion of exo-miR-224-3p. In the future, extensive studies on the mechanisms by which exosomal miRNAs, and even other noncoding RNAs, regulate lymphangiogenesis and LNM will be needed before exosomal vector-based tumor lymphangiogenesis therapies can be implemented in clinical practice.

## Conclusion

MiR-224-3p endocytosed by HLECs directly targets GSK3B mRNA 3′UTR in cells, inhibits GSK3B protein expression, and increases β-catenin stability. The activation of β-catenin in the nucleus enhances the expression of PROX1, thereby promoting tumor lymphangiogenesis. In addition, we also confirm that miR-224-3p is sorted into EVs by hnRNPA1 in GC cells, and secreted out. Our study reveals a new mechanism of lymphangiogenesis and LNM regulation, which provides a novel way for clinical intervention of lymph nodes in GC. The pattern diagram is shown in Fig. 8.

## Data Availability

All data that support the findings of this study are available from the corresponding author upon request.

## Abbreviations

GC: Gastric cancer
LNM: Lymph node metastasis
VEGFC: Vascular endothelial growth Factor C
RBPs: RNA-binding proteins
EVs: Extracellular vesicles
MVB: Multivesicular body
GSK-3: Glycogen synthase kinase-3
HC: Healthy control
qRT‒PCR: Quantitative real-time PCR
SDS: Sodium dodecyl sulfate
PBS: Phosphate-buffered saline
FBS: Fetal bovine serum
IHC: Immunohistochemistry
LVD: Lymphatic vessel density
HLECs: Human lymphatic endothelial cells
NC: Normal control
WT: Wild-type
MUT: Mutant type
WB: Western blot
YTHDC1: YTH Domain Containing 1
ACO1: Aconitase 1
hnRNPA1: Heterogeneous nuclear ribonucleoprotein A1
hnRNP: Heterogeneous nuclear ribonucleoprotein
